# A multi-ethnic breast cancer case–control study in New Zealand: evidence of differential risk patterns

**DOI:** 10.1007/s10552-012-0099-3

**Published:** 2012-11-21

**Authors:** Mona Jeffreys, Fiona McKenzie, Ridvan Firestone, Michelle Gray, Soo Cheng, Ate Moala, Neil Pearce, Lis Ellison-Loschmann

**Affiliations:** 1 School of Social and Community Medicine, University of Bristol, Canynge Hall, 39 Whatley Rd, Bristol, BS6 7TQ UK; 2 International Agency for Research on Cancer, Lyon, France; 3 Centre for Public Health Research, Massey University, Wellington, New Zealand; 4 Pacific Orthopaedics, Wellington, New Zealand; 5 London School of Hygiene and Tropical Medicine, Keppel Street, London, WC1E 7HT UK

**Keywords:** Breast cancer, Multi-ethnic, Case–control study, New Zealand

## Abstract

**Purpose:**

To investigate whether the relationships between established risk factors and breast cancer risk differ between three ethnic groups in New Zealand, namely Māori, Pacific, and non-Māori/non-Pacific women.

**Methods:**

The study is a multi-ethnic, age-, and ethnicity-matched population-based case–control study of breast cancer in women. Women with a primary, invasive breast cancer registered on the New Zealand Cancer Registry between 1 April 2005 and 30 April 2006, and Māori or Pacific women diagnosed to 30 April 2007 were eligible. Control women were identified from the New Zealand Electoral Roll, stratified by ethnicity, then frequency matched on age to the cases. Logistic regression was used to estimate odds ratios (OR) and 95 % confidence intervals (CI) between exposures and breast cancer risk in three ethnic groups separately. Likelihood ratio tests were used to test for modification of the effects by ethnicity. Post-stratification weighting of the controls was used to account for differential non-response by deprivation category.

**Results:**

The study comprised 1,799 cases (302 Māori, 70 Pacific, 1,427 non-Māori/non-Pacific) and 2,543 controls (746 Māori, 194 Pacific, 1,603 non-Māori/non-Pacific), based on self-identified ethnicity. Māori women were more likely to have ER and PR positive breast cancer compared to other ethnicities. There were marked differences in exposure prevalence between ethnicities and some differing patterns of risk factors for breast cancer between the three main ethnic groups. Of interest was the strong relationship between number of children and lower breast cancer risk in Pacific women (OR for 4 or more vs. 1 child OR 0.13, 95 % CI 0.05–0.35) and a higher risk of breast cancer associated with smoking (OR 1.76, 95 % CI 1.25–2.48) and binge drinking (5 or more vs. 1–2 drinks per occasion, OR 1.55, 95 % CI 1.07–2.26) in Māori women. Some of the documented results were attenuated following post-stratification weighting.

**Conclusions:**

The findings of this study need to be interpreted with caution, given the possibility of selection bias due to low response rates among some groups of women. Reducing the burden of breast cancer in New Zealand is likely to require different approaches for different ethnic groups.

## Introduction

The burden of breast cancer in both developed and developing countries is high and continues to rise. A large body of evidence of risk factors for breast cancer exists internationally. The most clearly established risk factors are reproductive variables [1], for example early menarche, late age at first birth, low parity, and late menopause, which are not amenable to intervention. Lifestyle factors which have been related to breast cancer in observational studies include alcohol consumption, low levels of physical activity, and smoking [2–4]. The multi-ethnic nature of the New Zealand population, with different ethnic groups exhibiting differences in breast cancer risk and different risk factor profiles, provides an opportunity for further exploration of some of these issues.

Māori are the indigenous people of New Zealand, comprising about 15 % of the population. Pacific people, who have migrated from small Pacific islands (e.g., Samoa, Cook Islands, Tonga, and Niue), comprise a further 7 %. Migration began as early as the late 1800s, but the majority of migration of Pacific people to New Zealand occurred in the 1960s and 1970s. The remainder of the population is composed primarily of people, originating from the United Kingdom and Europe (77 %), with a further 10 % of peoples from Asian countries (South East Asian, Chinese, Indian, and Other Asian). These last two groups are hereafter referred to as non-Māori/non-Pacific. The figures quoted here add up to over 100 %, since people who identify with more than one ethnicity are included in each ethnic population with which they identify [5].

Evidence regarding ethnic differences in breast cancer rates in New Zealand is mixed, because of changes in how ethnicity is measured, as well as changes in incidence over time. Older data for Māori suggest that their higher risk is restricted to those who identify as solely Māori, but is not apparent in the total Māori population [6]. More recent data are indicative of a higher risk compared to European/Other New Zealanders in the total Māori population, based on self-identified ethnicity [7], as well as an “ever-Māori” indicator [8], based on ethnicity as reported in electronic health records. The incidence of breast cancer in Māori women appears to be increasing faster than that in other ethnicities, with age-standardized rate differences between Māori and European/Other women having increased from 8 per 100,000 in 1981–1986 to 39 per 100,000 in 2001–2004 [7]. Overall, Pacific women living in New Zealand do not appear to have a higher risk than non-Māori/non-Pacific women [9, 10], although recent data suggest that young Pacific women (under 45 years) have a higher risk of breast cancer than European/Other New Zealanders, whereas older Pacific women (over 65 years) have a lower risk [7]. These differing patterns of breast cancer risk are not easily explained by known risk factor distributions [7].

Epidemiological studies of causal effects are limited by issues of confounding. Since most studies of breast cancer risk factors have been undertaken in countries where confounding structures are similar, it is not possible to know whether repeatedly observed associations are causal, or whether they are due to confounding. Alternative methods to address these issues have been proposed, including comparison of results from populations where confounding structures may differ [11]. New Zealand is a multicultural society, and confounding structures between important breast cancer risk factors may differ between ethnic groups, as well as differing from those in other developed countries. We have therefore used this opportunity to explore whether lifestyle factors affect breast cancer risk differentially between different ethnic groups.

The overall aim of the study was to explore the relationship between health behaviors across the lifecourse and breast cancer risk in three ethnic groups (Māori, Pacific and non-Māori/non-Pacific). This initial paper describes the methods used, response rates obtained, and demographics of the participants in the study and explores whether the relationships between established risk factors and breast cancer differ between three ethnic groups in New Zealand.

## Methods

The study is a multi-ethnic, age-, and ethnicity-matched population-based case–control study of breast cancer in women, with over-sampling of Māori and Pacific women, to ensure sufficient statistical power for most ethnic-specific analyses.

### Pilot study

To address issues of likely response rates and acceptability of the questionnaire content, a pilot study of 15 cases and 15 controls was conducted between November 2003 and February 2004. Cases were identified from the New Zealand Cancer Registry (NZCR); controls were selected from women on the New Zealand Electoral Roll, as described in detail under the main study, below. The pilot study identified that this method was inappropriate for Pacific women, most probably due to their higher levels of mobility and English as a second language. We therefore expanded our methods for recruitment of Pacific women controls in the main study. No issues of unacceptability or other methodological problems were identified during the pilot study.

### Main study

All women with a primary, invasive breast cancer (ICD10 C50.0-C50.9), registered on the NZCR between 1 April 2005 and 30 April 2006, were eligible for inclusion. In addition, to ensure sufficient numbers of Māori and Pacific women, cases who were identified on the Registry as being of Māori or Pacific ethnicity, diagnosed between 1 April 2006 and 30 April 2007 were eligible. Using data on the NZCR, the facility where the woman had been diagnosed was identified, and the Clinical Records Department (CRD) of that hospital was contacted, to ask for details of the woman’s GP. GPs were contacted, to ask if they knew of any reason why the woman should not be contacted, and if not, to supply contact details for the woman. Two attempts were made to contact each GP. Following this, each woman was contacted by post, followed by a reminder letter if no reply was received. The cases completed their questionnaires between January 2006 and December 2008.

Control women were stratified by ethnicity, then frequency matched on age, based on the expected age distribution in the cases from previously published incidence data, using 5-year age bands. The main method to identify population-based controls was through the electoral roll, registration for which is mandatory in New Zealand. There are two electoral rolls, “General” and “Māori.” Māori people can choose the electoral roll on which they want to be included. All people on the General Electoral Roll are asked to self-identify whether or not they are Māori or the descendent of a Māori.

Throughout the control selection process, which took place between November 2005 and October 2009, the most recent of the 2005, 2006, or 2008 Electoral Rolls was used. Māori women were randomly chosen in equal numbers from the General and Māori Electoral Roll, using the “Of Māori descent” indicator on the General Electoral Roll to identify Māori. For Pacific women, it was originally planned to use the General Electoral Roll to identify population-based controls. The first, middle, and last names of all women were searched on the General Electoral Roll, to identify whether these women were likely to be of Pacific ethnicity. Given that the majority of Pacific women in the target age group would be first generation migrants, we expected that the validity of this method would be relatively high. However, the response rate to this method was low (see below). Therefore, this was supplemented through two methods: (1) GPs of Pacific cases were invited to identify one of their patients, matched by age to the case at their practice, who: self-identified as being of Pacific ethnicity; had never had a diagnosis of breast cancer, and were not too ill to participate (2) controls were selected by trained Pacific nurses working at the Pacific Community Health Services from the main District Health Board (DHB) areas, namely Auckland, Wellington, and Canterbury. The nurses identified eligible controls on their current case list using the same criterion listed above for GPs and invited them to participate. For non-Māori/non-Pacific women, controls were identified from the General Electoral Roll among those eligible to act as controls, that is, based on the age distribution of cases, without the “Of Māori descent” indicator, and without a Pacific-sounding name.

### Exposure measurement

All women were given the option of completing the questionnaire at home and returning it by post, or by completing it over the telephone. In the latter case, the participant had a copy of the questionnaire in front of her, while she answered the questions which were asked by the trained interviewer. Māori and Pacific women were also given the option of face-to-face interviews. All study materials were translated into Samoan and Tongan (the most commonly spoken Pacific languages in New Zealand) and provided to the participants on request. Face-to-face and telephone interviews were conducted in the language of choice of the interviewee.

The questionnaire comprised sections on socio-demographics, childhood exposures, lifecourse exposures to health behaviors, and comprehensive occupational and reproductive histories. For current exposures, both cases and controls were asked to report their lifestyles 1 year previously. Control women were asked about attendance at screening, and cases were asked about their route to diagnosis and experiences associated with that process. Where possible, validated questions were used [12–14], and for some exposures, questions were based on previously used questionnaires. Women were asked to report their weight and height, from which body mass index (BMI) was determined. A disposable tape measure was sent with the questionnaire booklet, and participants were given instructions about how to measure their waist and hip circumferences, from which waist–hip ratio (WHR) was determined. Waist–height ratio (WHtR) was similarly calculated from reported measures. A copy of the questionnaire is available from the authors on request.

Age was defined as age at diagnosis for the cases and age at interview for the controls. Due to an error in the questionnaire, age at menopause could not be determined for the majority of women. The following rules were used to determine pre-/postmenopausal status at the time of diagnosis for the cases and at the time of questionnaire completion for the controls. Women were classified as premenopausal if they had a menstrual period in the last 3 months, or if their periods had stopped due to pregnancy/lactation, or use of hormonal birth control. Women were classified as postmenopausal if they reported natural menopause, surgical menopause involving bilateral oophorectomy, or use of hormone replacement therapy (HRT). Women who did not fall into these categories, who reported surgical menopause without bilateral oophorectomy, and other or unknown reasons for menses cessation were classified in an “other amenorrhea” category. This category was then dichotomized for analysis; women less than 49 years were considered premenopausal (*n* = 118) and women of 49 years and older were considered postmenopausal (*n* = 490). The cut off of 49 years was used as this is the median age at menopause reported in recent UK and New Zealand data [15, 16].

Deprivation was assessed using the NZDep2006 measure [17], an area-based measure derived from the 2006 census variables, based on place of residence at the time of diagnosis of the case and the time of interview of the control. Educational achievement was grouped into whether a woman had a postschool qualification, a school qualification only, or no qualifications. Therefore, women who had left school with no qualifications but subsequently obtained a postschool qualification were categorized in the most educated group. Exercise was assessed using the Godin questionnaire [12], based on self-reported exercise frequency and intensity, which were combined according to the recommended algorithm and analyzed in quartiles.

### Statistical analysis

Ethnicity data were coded using a prioritized system, which assigns people to a single, mutually exclusive category based on an established (Māori, Pacific, non-Māori/non-Pacific) hierarchy [18]. During the recruitment phase of the study, ethnicity was based on that recorded on the NZCR or on the Electoral Roll. Unweighted kappa statistics were used to assess agreement between recorded and self-reported ethnicity, as reported in the questionnaire. All further analyses were then based on self-reported ethnicity, as is standard practice in New Zealand health research.

Continuous variables were categorized using pre-defined cut-offs or quantiles; Chi squared tests were used to compare variable distributions across ethnic groups. Logistic regression was used to estimate odds ratios (OR) and 95 % confidence intervals (CI) adjusted for age group (four categories) and menopausal status at diagnosis. Analyses were reported stratified by ethnic group. Where effects were found, exploratory analyses were conducted, stratifying results by menopausal status, and/or adjusting for likely confounders. These results were presented in the text rather than tables. In addition to visual inspection of the ethnic-specific results, likelihood ratio tests were used as formal tests of interaction between explanatory variables and ethnicity. Due to the relatively small levels of missing data, these were excluded from all models, with the exception of nulliparous women in the analyses of age at first live birth and ever having breast-fed, in which case they were entered as a separate category.

Because of the low response rates in the control group (see below) and the evidence of differential non-response by deprivation quintile, we performed a sensitivity analysis to investigate non-response bias, using post-stratification weights. A weight was calculated for each stratum of ethnicity * deprivation, by dividing the expected deprivation distribution of each ethnic group by the observed deprivation distribution in the controls from our study. The expected distributions were estimated from the 2002/2003 New Zealand Health Survey (unpublished data) and were 2, 3, 10, 20, and 65 % for Māori and Pacific women in quintiles 1–5 of the NZDep2006 categories and 23, 20, 20, 20, and 17 % for non-Māori/non-Pacific women. Logistic regression models were then weighted using the “svy: logistic” command in Stata.

### Ethical approval

The pilot study was approved by the Wellington Ethics Committee, and the full study granted approval by the Multi-Region Ethics Committee (WGT/03/12/126).

## Results

### Cases

**Fig. 1:**
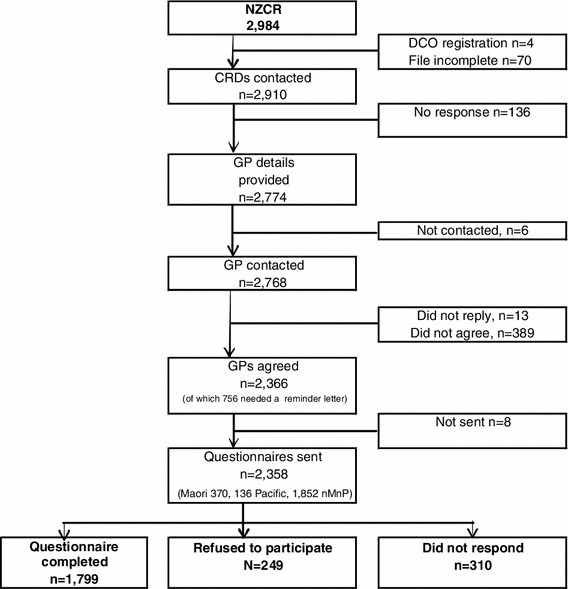
Response rates in cases

A total of 2,984 women with breast cancer were identified from the NZCR. Four women had a date of death on the same day as the date of diagnosis, and 70 women had incomplete files, so were not followed up. The remainder of CRDs, then GPs, was contacted. A total of 2,356 women were invited to take part, of whom 2,074 (88 %) responded, 1,869 (76 %) agreed to take part, and 1,799 women completed a questionnaire. Further details are shown in Fig. 1. Most questionnaires were self-completed (*n* = 1,612, 89 %); the remainder were completed over the telephone with an interviewer (*n* = 155, 9 %) or at a face-to-face interview (*n* = 32, 2 %). Based on the ethnicity from the NZCR, the response rates in cases were 81 % in Māori, 46 % in Pacific, and 78 % in non-Māori/non-Pacific women.

**Fig. 2:**
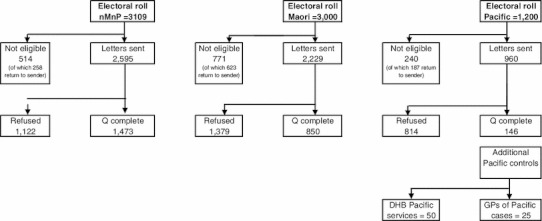
Response rates in controls. *Note*: The distributions by ethnicity given here are based on ethnicity as recorded in the electoral roll; numbers of participants according to self-identified ethnicity are given in the text. One of the Māori women who completed the questionnaire was subsequently excluded as she was found to be a transgender female

Details of recruitment of controls are shown in Fig. 2. A total of 3,109 non-Māori/non-Pacific women were identified from the General Electoral Roll. Of these, 258 (8 %) were returned to the study center as undelivered and 256 women who responded were not eligible (due to past breast cancer, having already died or being too ill to participate). Of the remaining 2,595 women, 1,473 (57 %) completed the questionnaire. One thousand five hundred Māori women were chosen from each of the General and Māori Electoral Rolls. Of the 3,000, 623 (21 %) were returned to the study center as undelivered. A further 148 women were not eligible. Of the remaining 2,229, 850 (38 %) women completed the questionnaire. We identified 1,200 women from the General Electoral Roll with a name that sounded of Pacific origin. Of these, 187 (16 %) were returned to the study center as undelivered and a further 53 (4 %) were not eligible. Of the remaining 960 women, 146 (15 %) were interviewed. Given the poor response rates, additional methods of identifying controls were employed (see above). This resulted in the recruitment of an additional 75 control women. In summary, the response rates among controls were 38 % in Māori, 15 % in Pacific women, and 57 % in non-Māori/non-Pacific women, based on the ethnicity data in the routinely collected data sources.

In summary, the study comprised 1,799 cases (302 Māori, 70 Pacific, 1,427 non-Māori/non-Pacific) and 2,543 controls (746 Māori, 194 Pacific, 1,603 non-Māori/non-Pacific), based on self-identified ethnicity. There was good agreement between the self-reported ethnicity and the data on ethnicity from the NZCR (cases), kappa = 88 %, and Electoral Roll (controls), kappa = 85 %. Among cases, the median time (inter quartile range) from diagnosis to interview was 8.4 months in non-Māori/non-Pacific women (7.3–11.4), 12.1 months in Māori women (8.6–19.1), and 15.1 months in Pacific women (11.7–19.2). The cases on the NZCR who took part were compared to those who did not take part. Non-Māori/non-Pacific and Pacific participants tended to be slightly younger than non-participants (median age 57.9 vs. 60.4 years for non-Māori/non-Pacific; 49.1 vs. 51.2 for Pacific cases), but no difference in median age between Māori participants and non-participants was evident (53.9 vs. 53.7 years).

Table 1 shows the distribution of the main breast cancer risk factors by ethnic group in cases and controls for completeness; discussion of the distribution of these risk factors between ethnic groups is restricted to the controls. It is noticeable that the greatest burden of breast cancer in the Pacific women in the study is in the under 50s, compared to the 50–65 year age band in Māori and non-Māori/non-Pacific women. Measures of socio-economic position show higher levels of deprivation and lower levels of education in Māori and Pacific women. The anthropometric measures show strong evidence of differences between ethnic groups. Māori and Pacific were taller, heavier and had higher WHR and WHtR, coupled with a high prevalence of self-reported diabetes. Maternal breast cancer was rarely reported by Pacific women.

**Table 1:** Distribution of known risk factors, among breast cancer cases and controls in New Zealand, stratified by self-identified ethnicity

	Cases				Controls			
	nMnP (*n* = 1,427)	Māori (*n* = 302)	Pacific (*n* = 70)	*p* value	nMnP (*n* = 1,603)	Māori (*n* = 746)	Pacific (*n* = 194)	*p* value
Age at interview								
20–35	25 (1.8)	10 (3.3)	4 (5.7)		19 (1.2)	46 (6.2)	23 (11.9)	
>35–50	377 (26.4)	97 (32.1)	37 (52.9)		334 (20.8)	289 (38.7)	73 (37.6)	
>50–65	589 (41.3)	137 (45.4)	19 (27.1)		767 (47.9)	337 (45.2)	72 (37.1)	
>65	436 (30.6)	58 (19.2)	10 (14.3)	<0.001	483 (30.1)	74 (9.9)	23 (11.9)	<0.001
Missing	–	–	–		–	–	3 (1.5)	
Interview method								
Phone	106 (7.4)	47 (15.6)	2 (2.9)		207 (12.9)	65 (8.7)	9 (4.6)	
Face	3 (0.2)	19 (6.3)	10 (14.3)		0 (0.3)	8 (1.1)	16 (8.3)	
Post	1,318 (92.4)	236 (78.2)	58 (82.9)	<0.001	1,396 (87.1)	673 (90.2)	169 (87.1)	<0.001
Deprivation quintile								
1–2	282 (19.8)	16 (5.3)	5 (7.1)		419 (26.1)	80 (10.7)	9 (4.6)	
3–4	265 (18.6)	31 (10.3)	5 (7.1)		395 (24.6)	118 (15.8)	18 (9.3)	
5–6	333 (23.3)	52 (17.2)	9 (12.9)		333 (20.8)	132 (17.7)	21 (10.8)	
7–8	325 (22.8)	73 (24.2)	21 (30.0)		290 (18.1)	179 (24.0)	41 (21.1)	
9–10	218 (15.3)	130 (43.1)	30 (42.9)	<0.001	164 (10.2)	237 (31.8)	102 (52.6)	<0.001
Missing	4 (0.3)	–	–		2 (0.1)	–	3 (1.6)	
Maternal breast cancer								
Yes	167 (11.7)	28 (9.3)	3 (4.3)		101 (6.3)	37 (5.0)	4 (2.1)	
No	1,237 (86.7)	262 (86.8)	66 (94.3)	0.019	1,476 (92.1)	677 (90.8)	176 (90.8)	<0.001
Missing	23 (1.6)	12 (4.0)	1 (1.4)		26 (1.6)	32 (4.3)	14 (7.2)	
Highest attained qualification								
None	392 (27.5)	117 (38.7)	24 (34.3)		389 (24.3)	212 (28.4)	73 (37.6)	
School qualification	287 (20.1)	35 (11.6)	12 (17.1)		271 (16.9)	122 (16.4)	27 (13.9)	
Postschool qualification	745 (52.2)	150 (49.7)	33 (47.1)	<0.001	942 (58.8)	409 (54.8)	93 (47.9)	0.001
Missing	3 (0.2)	–	1 (1.4)		1 (0.1)	3 (0.4)	1 (0.5)	
Height (cm)								
<=160	402 (28.2)	94 (31.1)	21 (30.0)		441 (27.5)	209 (28.0)	55 (28.4)	
160.1–165	429 (30.1)	80 (26.5)	12 (17.1)		485 (30.3)	207 (27.8)	38 (19.6)	
165.1–170	326 (22.9)	60 (19.9)	16 (22.9)		371 (23.1)	154 (20.7)	43 (22.2)	
>170	259 (18.2)	65 (21.5)	17 (24.3)	0.001	297 (18.5)	166 (22.3)	49 (25.3)	<0.001
Missing	11 (0.8)	3 (1.0)	4 (5.7)		9 (0.6)	10 (1.3)	9 (4.6)	
BMI (kg/m^2^)								
<25	643 (45.1)	76 (25.2)	6 (8.6)		797 (49.7)	236 (31.6)	15 (7.7)	
25–30	445 (31.2)	76 (25.2)	18 (25.7)		445 (27.8)	203 (27.2)	44 (22.7)	
>30–40	282 (19.8)	109 (36.1)	22 (31.4)		292 (18.2)	212 (28.4)	83 (42.8)	
>40	25 (1.8)	34 (11.3)	19 (27.1)	<0.001	33 (2.1)	61 (8.2)	35 (18.0)	<0.001
Missing	32 (2.2)	7 (2.3)	5 (7.1)		36 (2.3)	34 (4.6)	17 (8.8)	
Waist–hip ratio (tertiles)								
Min to 0.81	490 (34.3)	54 (17.9)	10 (14.3)		620 (38.7)	200 (26.8)	24 (12.4)	
0.81–0.87	502 (35.2)	85 (28.2)	10 (14.3)		536 (33.4)	224 (30.0)	38 (19.6)	
0.871 to max	394 (27.6)	145 (48.0)	44 (62.9)	<0.001	393 (24.5)	296 (39.7)	119 (61.3)	<0.001
Missing	41 (2.9)	18 (6.0)	6 (8.6)		54 (3.4)	26 (3.5)	13 (6.7)	
Waist–height ratio (tertiles)								
0.322–0.506	487 (34.1)	44 (14.6)	2 (2.9)		656 (40.9)	196 (26.3)	9 (4.6)	
0.506–0.584	511 (35.8)	84 (27.8)	13 (18.6)		532 (33.2)	206 (27.6)	26 (13.4)	
0.584–1.280	379 (26.6)	154 (51.0)	47 (67.1)	<0.001	353 (22.0)	311 (41.7)	139 (71.7)	<0.001
Missing	50 (3.5)	20 (6.6)	8 (11.4)		62 (3.9)	33 (4.4)	20 (10.3)	
Has had a diagnosis of diabetes								
Yes	115 (8.1)	58 (19.2)	12 (17.1)		102 (6.4)	84 (11.3)	37 (19.1)	
No	1,309 (91.7)	244 (80.8)	58 (82.9)	<0.001	1,498 (93.4)	659 (88.3)	156 (80.4)	<0.001
Missing	3 (0.2)	–			3 (0.2)	3 (0.4)	1 (0.5)	
Age at menarche								
<12	233 (16.3)	71 (23.5)	16 (22.9)		261 (16.3)	162 (21.7)	25 (12.9)	
12	338 (23.7)	73 (24.2)	9 (12.9)		354 (22.1)	203 (27.2)	41 (21.1)	
13	444 (31.1	81 (26.8)	18 (25.7)		499 (31.1)	184 (24.7)	48 (24.7)	
14+	387 (27.1)	70 (23.2)	26 (37.1)	0.017	472 (29.4)	189 (25.3)	75 (38.7)	<0.001
Missing	25 (1.8)	7 (2.3)	1 (1.4)		17 (1.1)	8 (1.1)	5 (2.6)	
Menopausal status at diagnosis/interview								
Premenopausal	413 (28.9)	95 (31.5)	38 (54.3)		392 (24.5)	332 (44.5)	95 (49.0)	
Postmenopausal	1,014 (71.1)	207 (68.5)	32 (45.7)	<0.001	1,211 (75.6)	414 (55.5)	99 (51.0)	<0.001
Ever used oral contraceptive								
Yes	1,058 (74.1)	202 (66.9)	33 (47.1)		1,282 (80.0)	592 (79.4)	80 (41.2)	
No	367 (25.7)	98 (32.5)	36 (51.4)	<0.001	318 (19.8)	147 (19.7)	112 (57.7)	<0.001
Missing	2 (0.1)	2 (0.7)	1 (1.4)		3 (0.2)	7 (0.9)	2 (1.0)	
Ever used HRT								
Yes	342 (24.0)	40 (13.3)	2 (2.9)		397 (24.8)	91 (12.2)	4 (2.1)	
No	1,068 (74.8)	260 (86.1)	68 (97.1)	<0.001	1,191 (74.3)	646 (86.6)	189 (97.4)	<0.001
Missing	17 (1.2)	2 (0.7)	0		15 (0.9)	9 (1.2)	1 (0.5)	
Number of live births								
0	185 (13.0)	34 (11.3)	15 (21.4)		154 (9.6)	79 (10.6)	30 (15.5)	
1	142 (10.0)	43 (14.2)	13 (18.6)		135 (8.4)	85 (11.4)	16 (8.3)	
2	465 (32.6)	61 (20.2)	14 (20.0)		548 (34.2)	182 (24.4)	31 (16.0)	
3	364 (25.5)	55 (18.2)	16 (22.9)		448 (28.0)	196 (26.3)	25 (12.9)	
4+	266 (18.6)	107 (35.4)	11 (15.7)	<0.001	311 (19.4)	200 (26.8)	88 (45.4)	<0.001
Missing	5 (0.4)	2 (0.7)	1 (1.4)		7 (0.4)	4 (0.5)	4 (2.1)	
Ever breastfed								
Yes	1,045 (73.2)	226 (74.8)	47 (67.1)		1,244 (77.6)	563 (75.5)	143 (73.7)	
No	188 (13.2)	37 (12.3)	7 (10.0)		197 (12.3)	98 (13.1)	15 (7.7)	
Nulliparous	185 (13.0)	34 (11.3)	15 (21.4)		154 (9.6)	79 (10.6)	30 (15.5)	
Missing	9 (0.6)	5 (1.7)	1 (1.4)	0.17	8 (0.5)	6 (0.8)	6 (3.1)	<0.001
Age at first live birth								
Nulliparous	185 (13.0)	34 (11.3)	15 (21.4)		154 (9.6)	79 (10.6)	30 (15.5)	
<20	159 (11.1)	97 (32.1)	11 (15.7)		181(11.3)	224 (30.0)	29 (15.0)	
20–24	533 (37.4)	105 (34.8)	16 (22.9)		585 (36.5)	250 (33.5)	73 (37.6)	
25–29	345 (24.2)	42 (13.9)	15 (21.4)		414 (25.8)	110 (14.8)	33 (17.0)	
>=30	195 (13.7)	20 (6.6)	10 (14.3)	<0.001	254 (15.9)	69 (9.3)	17 (8.8)	<0.001
Missing	10 (0.7)	4 (1.3)	3 (4.3)		15 (0.9)	14 (1.9)	12 (6.2)	
Total duration of breast feeding (parous women)								
Never	188 (15.1)	37 (13.8)	7 (12.7)		197 (13.6)	98 (14.7)	15 (9.2)	
Up to 6 months	276 (22.2)	53 (19.8)	7 (12.7)		280 (19.3)	99 (14.8)	13 (7.9)	
6–12 months	219 (17.6)	32 (11.9)	10 (18.2)		278 (19.2)	101 (15.1)	17 (10.4)	
Over 12 months	550 (44.3)	141 (52.6)	30 (54.6)	0.058	686 (47.3)	363 (54.4)	113 (68.9)	<0.001
Missing	9 (0.7)	5 (1.9)	1 (1.8)		8 (0.6)	6 (0.9)	6 (3.7)	
Ever smoked								
Yes	656 (46.0)	246 (81.5)	40 (57.1)		731 (45.6)	535 (71.7)	88 (45.4)	
No	771 (54.0)	56 (18.5)	29 (41.4)	<0.001	869 (54.2)	209 (28.0)	104 (53.6)	<0.001
Missing	–	–	1 (1.4)		4 (0.2)	2 (0.3)	2 (1.0)	
Frequency of alcohol consumption								
Never	253 (17.7)	81 (26.8)	37 (52.9)		227 (14.2)	149 (20.0)	109 (56.2)	
Monthly	339 (23.7)	100 (33.1)	16 (22.9)		343 (21.4)	241 (32.3)	50 (25.8)	
2–4/month	240 (16.8)	64 (21.2)	9 (12.9)		312 (19.5)	141 (18.9)	18 (9.3)	
2 or more per week	592 (41.5)	57 (18.9)	8 (11.4)	<0.001	721 (45.0)	210 (28.2)	16 (8.2)	<0.001
Missing	3 (0.2)					5 (0.7)	1 (0.5)	
Usual amount of alcohol consumed (drinks per occasion)								
0	253 (17.7)	81 (26.8)	37 (52.9)		227 (14.2)	149 (20.0)	109 (56.2)	
1–2	926 (64.9)	116 (38.4)	17 (26.3)		1,100 (68.6)	331 (44.4)	37 (19.1)	
3–4	158 (11.1)	35 (11.6)	7 (10.0)		196 (12.2)	121 (16.2)	22 (11.3)	
5+	51 (3.6)	66 (21.9)	9 (12.9)	<0.001	50 (3.1)	133 (17.8)	25 (12.9)	<0.001
Missing	39 (2.7)	4 (1.3)			30 (1.9)	12 (1.6)	1 (0.5)	
Exercise frequency and intensity (quartiles)								
1	371 (26.0)	111 (36.8)	27 (38.6)		357 (22.3)	221 (29.6)	39 (20.1)	
2	395 (27.7)	81 (26.8)	14 (20.0)		444 (27.7)	193 (25.9)	48 (24.7)	
3	293 (20.5)	46 (15.2)	7 (10.0)		371 (23.1)	132 (17.7)	34 (17.5)	
4	326 (22.9)	58 (19.2)	19 (27.1)	0.002	404 (25.2)	173 (23.2)	60 (30.9)	<0.001
Missing	42 (2.9)	6 (2.0)	3 (4.3)		27 (1.7)	27 (3.6)	13 (6.7)	

*nMnP* non-Māori/non-Pacific

* *p* values show differences between ethnic groups, omitting people with missing data

Reproductive variables differed by ethnic group. Mean age at menarche was lower in Māori (12.6 years) and non-Māori/non-Pacific (12.9 years) than Pacific (13.4 years) women. Greatest use of oral contraceptives and hormone replacement therapy (HRT) was reported in non-Māori/non-Pacific women, with lowest use in Pacific women. Over 45 % of Pacific controls reported having had four or more children, although it is noticeable that this pattern was not seen in the cases, see below for further discussion. Numbers of children were lower in Māori and non-Māori/non-Pacific women; Māori women reported the greatest proportion of births under age 20. Non-Māori/non-Pacific women were more likely than other ethnic groups to have ever breast-fed, but the total duration of breast feeding was highest in Pacific women.

Māori women reported a very high prevalence of ever having smoked. Over half of Pacific women reported never drinking alcohol; the majority of Māori and non-Māori/non-Pacific women were light drinkers. However, Māori women who did drink alcohol reported that they drank a higher number of drinks per occasion than other ethnic groups. Māori women appeared to be less active and Pacific women more active than non-Māori/non-Pacific women.

Over 98 % of cases were histologically confirmed, and this proportion did not differ by ethnicity. Further details of the cancers in the cases are given in Table 2. Among the respondents, there was no significant difference in the stage at presentation by ethnic group, but a high proportion of Pacific women were recorded as having unknown stage. The distribution by grade was similar between ethnic groups, as was the proportion of cancers that were ER positive. Māori women were more likely than the other ethnic groups to have PR positive breast cancer; Māori and Pacific women were more likely than non-Māori/non-Pacific women to have HER2 positive breast cancer, although the high degree of missing data for these latter analyses means that the results should be interpreted with caution. Among the women with a recorded ER, PR, and HER2 status, 124 were negative for all three (“triple negative breast cancer”). This was most common in non-Māori/non-Pacific women (13 %), compared to 4 % in Māori and 11 % in Pacific women, *p* = 0.002.

**Table 2:** Tumor characteristics of breast cancer cases in New Zealand

	nMnP (*n* = 1,427)	Māori (*n* = 302)	Pacific (*n* = 70)	*p* value*
Stage				
Local	712 (49.9)	136 (45.0)	28 (40.0)	
Regional	528 (37.0)	116 (38.4)	23 (32.9)	
Distant	16 (1.1)	7 (2.3)	2 (2.9)	0.24
Unknown	171 (12.0)	43 (14.2)	17 (24.3)	
Grade				
Well differentiated	348 (24.4)	68 (22.5)	14 (20.0)	
Moderately differentiated	587 (41.1)	134 (44.4)	31 (44.3)	
Poorly differentiated	417 (29.2)	81 (26.8)	20 (28.6)	0.73
Unknown	75 (5.3)	19 (6.3)	4 (7.1)	
ER positive				
Yes	1,075 (75.3)	240 (79.5)	46 (65.7)	
No	252 (17.7)	41 (13.6)	15 (21.4)	0.102
Unknown	100 (7.0)	21 (7.0)	9 (12.9)	
PR positive				
Yes	864 (60.6)	209 (69.2)	41 (58.6)	
No	455 (31.9)	64 (21.2)	20 (28.6)	0.002
Unknown	108 (7.6)	29 (9.6)	9 (12.9)	
HER2 positive				
Yes	134 (9.4)	47 (15.6)	10 (14.3)	
No	734 (51.4)	155 (51.3)	37 (52.9)	0.021
Unknown	559 (39.2)	100 (33.1)	23 (32.9)	
Tumor size (mm)				
<10	308 (21.6)	34 (11.3)	9 (12.9)	
10–19	416 (29.2)	69 (22.9)	16 (22.9)	
20–29	325 (22.8)	105 (34.8)	13 (18.6)	
30+	255 (17.9)	58 (19.2)	19 (27.1)	<0.001
Missing	123 (8.6)	36 (11.9)	13 (18.6)	

*nMnP* non-Māori/non-Pacific

* The *p* value shows the difference between ethnic groups, calculated after excluding missing data

Associations between “known” risk factors and breast cancer are shown in Table 3. In interpreting these data, it is important to acknowledge the low statistical power among Pacific women, and the large number of comparisons made, thus increasing the likelihood of chance findings. Furthermore, because of the evidence of differential non-response by deprivation category, the results need to be interpreted alongside those presented in Table 4, which have been weighted to account for this possible selection bias.

**Table 3:** Adjusted odds ratios (OR) and 95 % confidence intervals (CI) showing the association between known risk factors and breast cancer risk in three ethnic groups in New Zealand

	nMnP	Māori	Pacific
	OR (95 % CI)	OR (95 % CI)	OR (95 % CI)
Deprivation quintile			
1–2	1*	1*	1*
3–4	1.00 (0.80–1.24)	1.28 (0.65–2.51)	0.56 (0.12–2.51)
5–6	1.48 (1.19–1.84)	1.94 (1.03–3.65)	0.70 (0.18–2.78)
7–8	1.68 (1.35–2.10)	1.90 (1.03–3.51)	0.86 (0.25–3.00)
9–10	2.06 (1.59–2.66)	2.36 (1.31–4.26)	0.48 (0.14–1.60)
*p* (interaction)**	0.18		
Maternal breast cancer			
Yes	1.98 (1.52–2.56)	1.83 (1.07–3.12)	1.69 (0.35–8.15)
No	1*	1*	1*
*p* (interaction)		0.96	
Highest attained qualification			
None	1*	1*	1*
School qualification	0.98 (0.78–1.22)	0.69 (0.43–1.10)	1.35 (0.58–3.15)
Postschool qualification	0.72 (0.60–0.86)	0.84 (0.62–1.15)	1.16 (0.61–2.22)
*p* (interaction)		0.018	
Height (cm)			
<=160	1*	1*	1*
160.1–165	0.96 (0.80–1.16)	0.90 (0.63–1.30)	0.91 (0.39–2.14)
165.1–170	0.96 (0.78–1.17)	0.92 (0.61–1.37)	0.93 (0.42–2.06)
>170	0.92 (0.74–1.14)	1.00 (0.67–1.48)	0.77 (0.35–1.71)
*p* (interaction)		0.98	
BMI (kg/m^2^)			
<25	1*	1*	1*
25–30	1.29 (1.09–1.53)	1.10 (0.75–1.60)	1.22 (0.40–3.76)
>30–40	1.27 (1.04–1.55)	1.39 (0.97–1.99)	0.75 (0.25–2.20)
>40	0.98 (0.57–1.68)	1.58 (0.95–2.62)	1.71 (0.55–5.33)
*p* (interaction)		0.19	
BMI (kg/m^2^), premenopausal women only			
<25	1*	1*	1*
>=25	1.05 (0.79–1.40)	1.47 (0.88–2.46)	0.84 (0.21–3.28)
*p* (interaction)		0.37	
BMI (kg/m^2^), postmenopausal women only			
<25	1*	1*	1*
>=25	1.36 (1.15–1.62)	1.21 (0.82–1.80)	1.62 (0.32–8.62)
*p* (interaction)		0.94	
Waist–hip ratio (tertiles)			
1	1*	1*	1*
2	1.21 (1.02–1.44)	1.29 (0.86–1.92)	0.56 (0.20–1.59)
3	1.30 (1.08–1.57)	1.56 (1.08–2.26)	0.80 (0.35–1.85)
*p* (interaction)		0.28	
Waist–height ratio (tertiles)			
1	1*	1*	1*
2	1.37 (1.15–1.63)	1.58 (1.04–2.42)	2.40 (0.44–13.17)
3	1.54 (1.27–1.86)	1.82 (1.23–2.69)	1.57 (0.32–7.73)
*p* (interaction)		0.23	
Has had a diagnosis of diabetes			
Yes	1.35 (1.02–1.79)	1.51 (1.03–2.22)	0.85 (0.41–1.78)
No	1*	1*	1*
*p* (interaction)		0.13	
Age at menarche			
<12	1.00 (0.80–1.24)	0.96 (0.65–1.43)	1.82 (0.76–4.32)
12	1.07 (0.88–1.30)	0.81 (0.55–1.19)	0.50 (0.20–1.27)
13	1*	1*	1*
14+	0.91 (0.76–1.10)	0.83 (0.57–1.23)	1.01 (0.49–2.11)
*p* (interaction)		0.23	
Ever used oral contraceptive			
Yes	0.67 (0.56–0.81)	0.65 (0.47–0.90)	1.19 (0.66–2.13)
No	1*	1*	1*
*p* (interaction)		0.014	
Ever used HRT			
Yes	1.08 (0.91–1.30)	0.98 (0.64–1.50)	1.89 (0.31–11.33)
No	1*	1*	1*
*p* (interaction)		0.70	
Number of live births			
0	1.17 (0.85–1.62)	0.99 (0.57–1.74)	0.67 (0.24–1.83)
1	1*	1*	1*
2	0.83 (0.64–1.09)	0.73 (0.45–1.19)	0.46 (0.17–1.28)
3	0.82 (0.62–1.08)	0.59 (0.36–0.95)	0.70 (0.26–1.92)
4+	0.86 (0.64–1.15)	0.92 (0.59–1.45)	0.13 (0.05–0.35)
*p* (interaction)		<0.001	
Age at first live birth			
Nulliparous	1.25 (0.92–1.70)	1.19 (0.73–1.94)	1.52 (0.56–4.15)
<20	1*	1*	1*
20–24	1.00 (0.78–1.28)	0.93 (0.66–1.31)	0.51 (0.20–1.29)
25–29	0.86 (0.66–1.12)	0.99 (0.63–1.53)	1.12 (0.43–2.96)
>=30	0.76 (0.57–1.01)	0.76 (0.43–1.34)	1.25 (0.43–3.69)
*p* (interaction)		0.28	
Ever breastfed			
Yes	0.85 (0.68–1.06)	1.16 (0.76–1.78)	0.62 (0.23–1.71)
No	1*	1*	1*
Nulliparous	1.20 (0.89–1.62)	1.46 (0.82–2.60)	1.17 (0.37–3.72)
*p* (interaction)		0.71	
Total duration of breast feeding (among parous women)			
Never	0.98 (0.75–1.28)	0.67 (0.40–1.12)	1.09 (0.28–4.21)
Up to 6 months	1*	1*	1*
6–12 months	0.78 (0.61–1.00)	0.60 (0.35–1.01)	1.18 (0.34–4.09)
Over 12 months	0.77 (0.63–0.94)	0.73 (0.49–1.09)	0.55 (0.20–1.56)
*p* (interaction)		0.54	
Ever smoked			
Yes	1.01 (0.88–1.17)	1.76 (1.25–2.48)	1.60 (0.90–2.83)
No	1*	1*	1*
*p* (interaction)		0.006	
Frequency of alcohol consumption			
Never	1.13 (0.89–1.43)	1.08 (0.74–1.57)	1.01 (0.50–2.06)
≤ Monthly	1*	1*	1*
2–4 drinks/month	0.75 (0.60–0.95)	1.21 (0.82–1.78)	1.39 (0.51–3.82)
2 or more drinks/wk	0.81 (0.67–0.98)	0.70 (0.47–1.02)	1.47 (0.52–4.16)
*p* (interaction)		0.082	
Usual amount of alcohol consumed (drinks per occasion)			
0	1.33 (1.08–1.63)	1.24 (0.87–1.78)	0.76 (0.37–1.56)
1–2	1*	1*	1*
3–4	0.90 (0.72–1.14)	0.86 (0.56–1.34)	0.72 (0.25–2.06)
5+	1.12 (0.74–1.67)	1.55 (1.07–2.26)	0.83 (0.31–2.22)
*p* (interaction)		0.53	
Exercise frequency and intensity (quartiles)			
1	1*	1*	1*
2	0.88 (0.72–1.08)	0.89 (0.63–1.28)	0.44 (0.20–0.97)
3	0.77 (0.62–0.95)	0.80 (0.53–1.22)	0.32 (0.12–0.86)
4	0.80 (0.65–1.00)	0.77 (0.52–1.13)	0.43 (0.21–0.91)
*p* (interaction)		0.53	

OR are adjusted for age, menopausal status at diagnosis and interview method (postal, telephone or face-to-face interviews)

*nMnP* non-Māori/non-Pacific

* Reference category

** Test of interaction of differences in OR between ethnic groups

**Table 4:** Weighted, adjusted Odds Ratios (OR) and 95 % confidence intervals (CI) showing the association between known risk factors and breast cancer risk in three ethnic groups in New Zealand, weighted using post-stratification weights to account for differential non-response bias by deprivation quintile

	nMnP	Māori	Pacific
	OR (95 % CI)	OR (95 % CI)	OR (95 % CI)
Deprivation quintile			
1–2	1*	1*	1*
3–4	1.02 (0.86–1.21)	1.88 (1.02–3.48)	1.51 (0.37–6.19)
5–6	1.07 (0.91–1.26)	1.08 (0.61–1.92)	0.68 (0.17–2.74)
7–8	0.92 (0.78–1.08)	0.94 (0.54–1.64)	1.65 (0.49–5.49)
9–10	0.42 (0.35–0.51)	0.64 (0.38–1.10)	1.68 (0.51–5.57)
Highest attained qualification			
None	1*	1*	1*
School qualification	1.13 (0.88–1.45)	0.82 (0.50–1.34)	1.06 (0.44–2.60)
Postschool qualification	0.89 (0.73–1.08)	0.97 (0.70–1.36)	0.97 (0.47–2.01)
Height (cm)			
<=160	1*	1*	1*
160.1–165	1.05 (0.85–1.30)	1.02 (0.70–1.52)	0.83 (0.32–2.10)
165.1–170	1.09 (0.87–1.36)	1.04 (0.68–1.65)	1.19 (0.52–2.74)
>170	1.01 (0.79–1.30)	1.04 (0.67–1.59)	0.99 (0.40–2.18)
BMI (kg/m^2^), premenopausal women only			
<25	1*	1*	1*
>=25	1.12 (0.82–1.55)	1.45 (0.83–2.51)	0.95 (0.23–3.92)
BMI (kg/m^2^), postmenopausal women only			
<25	1*	1*	1*
>=25	1.29 (1.06–1.56)	0.99 (0.64–1.52)	2.23 (0.39–12.67)
Waist–hip ratio (tertiles)			
1	1*	1*	1*
2	1.18 (0.97–1.43)	1.23 (0.79–1.92)	0.51 (0.15–1.75)
3	1.21 (0.97–1.49)	1.27 (0.83–1.93)	0.94 (0.34–2.58)
Waist–height ratio (tertiles)			
1	1*	1*	1*
2	1.26 (1.03–1.54)	1.41 (0.89–2.24)	1.52 (0.24–9.68)
3	1.38 (1.11–1.71)	1.47 (0.97–2.23)	1.37 (0.25–7.58)
Has had a diagnosis of diabetes			
Yes	1.25 (0.91–1.72)	1.43 (0.96–2.14)	0.91 (0.39–2.11)
No	1*	1*	1*
Age at menarche			
<12	0.90 (0.70–1.15)	0.98 (0.65–1.50)	1.71 (0.66–4.39)
12	0.96 (0.77–1.21)	0.82 (0.54–1.25)	0.44 (0.16–1.26)
13	1*	1*	1*
14+	0.81 (0.66–1.00)	0.81 (0.53–1.22)	1.05 (0.47–2.31)
Ever used oral contraceptive			
Yes	0.78 (0.64–0.96)	0.80 (0.57–1.12)	1.06 (0.56–2.02)
No	1*	1*	1*
Ever used HRT			
Yes	1.18 (0.97–1.44)	1.09 (0.69–1.71)	0.91 (0.11–7.21)
No	1*	1*	1*
Number of live births			
0	1.10 (0.76–1.57)	1.07 (0.59–1.94)	0.74 (0.23–2.34)
1	1*	1*	1*
2	0.84 (0.63–1.13)	0.77 (0.46–1.29)	0.65 (0.21–2.03)
3	0.78 (0.58–1.06)	0.54 (0.32–0.90)	0.80 (0.25–2.53)
4+	0.74 (0.54–1.03)	0.84 (0.52–1.36)	0.18 (0.06–0.53)
Age at first live birth			
Nulliparous	1.48 (1.04–2.12)	1.45 (0.86–2.45)	1.10 (0.35–3.44)
<20	1*	1*	1*
20–24	1.17 (0.89–1.56)	1.03 (0.71–1.49)	0.40 (0.14–1.13)
25–29	1.10 (0.82–1.48)	1.09 (0.68–1.74)	0.94 (0.31–2.83)
>=30	1.12 (0.81–1.54)	1.02 (0.54–1.93)	1.18 (0.36–3.82)
Ever breastfed			
Yes	0.97 (0.76–1.25)	1.16 (0.74–1.82)	0.71 (0.24–2.14)
No	1*	1*	1*
Nulliparous	1.31 (0.92–1.85)	1.63 (0.87–3.05)	1.10 (0.29–4.15)
Total duration of breast feeding (among parous women)			
Never	0.88 (0.63–1.24)	0.67 (0.37–1.21)	1.39 (0.27–7.11)
Up to 6 months	1*	1*	1*
6–12 months	0.75 (0.55–1.03)	0.62 (0.34–1.12)	1.54 (0.27–8.86)
Over 12 months	0.83 (0.64–1.08)	0.69 (0.44–1.09)	0.75 (0.19–2.99)
Ever smoked			
Yes	0.96 (0.82–1.12)	1.40 (0.98–2.01)	1.73 (0.94–3.17)
No	1*	1*	1*
Frequency of alcohol consumption			
Never	1.10 (0.84–1.44)	1.00 (0.66–1.49)	1.05 (0.47–2.35)
≤ Monthly	1*	1*	1*
2–4 drinks/month	0.91 (0.70–1.16)	1.30 (0.87–1.95)	1.18 (0.41–3.37)
2 or more drinks/wk	1.00 (0.81–1.23)	0.87 (0.58–1.29)	1.29 (0.41–4.02)
Usual amount of alcohol consumed (drinks per occasion)			
0	1.09 (0.86–1.38)	0.94 (0.63–1.39)	0.81 (0.36–1.45)
1–2	1*	1*	1*
3–4	0.85 (0.66–1.10)	0.68 (0.43–1.10)	0.65 (0.21–1.99)
5+	0.88 (0.56–1.38)	1.23 (0.83–1.81)	0.85 (0.29–2.49)
Exercise frequency and intensity (quartiles)			
1	1*	1*	1*
2	1.01 (0.81–1.27)	0.99 (0.68–1.46)	0.39 (0.16–0.97)
3	0.94 (0.74–1.20)	0.86 (0.55–1.36)	0.29 (0.09–0.88)
4	0.94 (0.75–1.21)	0.84 (0.56–1.26)	0.35 (0.14–0.84)

OR are adjusted for age, menopausal status at diagnosis and interview method (postal, telephone, or face-to-face interviews)

* Reference category

Although for some risk factors, the association with breast cancer was similar between ethnic groups, for others, there were interesting patterns of difference. For anthropometric variables, Māori and non-Māori/non-Pacific women had similar patterns of associations, with women reporting a higher BMI, a higher WHR, or diabetes having a higher risk of breast cancer than women without those risk factors. The effect of weighting the controls for differential non-response attenuated many of these relationships. Restricting the results to women who were interviewed within 1 year of diagnosis did not change the effect of BMI on postmenopausal non-Māori/non-Pacific women, but attenuated the effect in postmenopausal Māori women.

The overall patterns were less clear for Pacific women. However, when Pacific women were restricted to those who were postmenopausal at diagnosis/interview, there was a suggestion of a positive association between BMI and breast cancer (OR per 5 kg/m^2^ 1.19, 95 % CI 0.92–1.52), which was not apparent for premenopausal breast cancer (OR per 5 kg/m^2^ 0.92, 95 % CI 0.69–1.22). When stratifying the Pacific women by menopausal status, the effect of WHR tertile and diagnosis of diabetes were both stronger in postmenopausal women, but none of the effects approached statistical significance. Analyzing the WHtR as a dichotomous variable, split at 0.5, gave similar patterns of associations as were seen for other measures of obesity; the OR were 1.37 (95 % CI 1.17–1.61) in non-Māori/non-Pacific women, 1.64 (95 % CI 1.12–2.42) in Māori women, and 1.47 (95 % CI 0.29–7.30) in Pacific women.

There was an unexpected protective effect of OC use on breast cancer, evident in Māori and non-Māori/non-Pacific women. This effect was unchanged following adjustment for BMI, deprivation, and number of live births. Among Māori women, duration of breast feeding explained part of the protective effect of OC use (adjusted OR 0.73, 95 % CI 0.50–1.06). On stratification, the effect was apparent for postmenopausal not premenopausal non-Māori/non-Pacific women (adjusted OR 0.60 95 % CI 0.48–0.75). The effect was weaker in the weighted analysis. No clear relationship between ever use of HRT and breast cancer was detected, although in non-Māori/non-Pacific women, the expected increased risk was apparent, having weighted for differential non-response in the controls.

Evidence for an expected protective effect of later age at menarche was weak in all ethnic groups. The protective effect of having more children and ever having breast-fed was particularly strong in Pacific women; age at first birth was not strongly related to breast cancer risk in this study, although there was a suggestion of a lower risk of breast cancer among women who had had their first child over the age of 30 years. Longer duration of breast feeding was associated with a lower risk of breast cancer in all ethnic groups.

Although smoking is not traditionally thought of as a “known” breast cancer risk factor, it was included in our analysis because of its importance for Māori public health. There was clear evidence for an interaction with ethnicity, with Māori women who had ever smoked having a 76 % higher risk of breast cancer than those who had never smoked. This was only partly explained by levels of deprivation and education; further adjustment for these factors only reduced the OR to 1.62 (95 % CI 1.14–2.30). The effect in Māori women was attenuated when weighted for differential non-response by deprivation level; in Pacific women, the effect, although unstable, showed a suggestion of a strengthening of effect. An estimation of the population attributable fraction for smoking and breast cancer in Māori based on this adjusted OR suggests that approximately 31 % of the burden of breast cancer in Māori women is attributable to smoking; however, this is likely to be an overestimate due to residual confounding and misclassification of deprivation and education.

Because of the relative rarity of frequent drinking, the two upper categories of frequency of alcohol consumption (“2–4 drinks” and “4 or more drinks” per week) were combined for analysis. There was weak evidence of an inverse association between frequency of alcohol consumption and breast cancer risk, although as shown in Table 4, this disappeared in the weighted analysis. In the unweighted analysis, the higher risk among women who were never drinkers compared to those who drank one to two drinks per occasion was eliminated in non-Māori/non-Pacific and Pacific women when the data were restricted to those who were interviewed within 1 year of diagnosis, but persisted in Māori women. In women of all ethnicities, exercise was associated with a lower risk of breast cancer. The results were not materially changed in any ethnic group when the data were restricted to those who were interviewed within 1 year of diagnosis.

## Discussion

The findings presented here report the first nationwide, population-based, multi-ethnic study of breast cancer in New Zealand. The results show differences in exposure prevalence between ethnicities and differing patterns of risk factors for breast cancer between the three main ethnic groups.

Many of the findings in this study are in agreement with those previously reported, but we have also highlighted some important differences. Some of the lack of associations in Pacific women in particular may be due to insufficient statistical power, since this was the smallest ethnic group studied. More generally, our study is affected by the limitations of case–control studies, particularly those involving interviews, principally the potential for selection and recall bias. Non-differential misclassification is also likely to have affected the results. To minimize selection bias, we attempted to maximize the response rate. Although this was good among cases (over 75 %), the response rates were poor among controls, ranging from 15 % in Pacific women to 59 % in non-Māori/non-Pacific women. The Pacific controls are therefore unlikely to be representative of the population of Pacific women in New Zealand. However, the paucity of research in this population group means that our work is the first documentation of breast cancer risk factors, measured on an individual level, in Pacific women.

If non-response is non-differential across determinants of breast cancer risk, the effect of the low response rates will simply be to reduce the numbers of women available for analysis, that is, the “non-responders” will be “missing” completely at random. However, this is unlikely to be the case. Although we did not have much information on the non-responders, we used the national distribution of the deprivation measure to investigate this further. Importantly, we found that our response rates were lower in women living in the most deprived areas, which may have biased the results based on markers of socio-economic position, as well as those which are socially patterned. The post-stratification weighting that we used was intended to address this. The greatest effect that the weighting had was on the effect of deprivation itself. For other factors, the weighting tended to dilute the observed effects. We therefore remain cautious in our interpretation of the results presented, given the potential for selection bias in our study.

Unlike any other ethnic group, the greatest burden of breast cancer in the Pacific women in the study is in the under 50s. This is consistent with data from Hawaii, where the “Asian/Pacific Islander” group is reported to have a lower age at diagnosis than white women [19, 20]. A recent report from New Zealand demonstrated higher rates of breast cancer in Pacific women in New Zealand aged under 45 compared to European New Zealanders, and lower rates of Pacific women aged over 65 years [7]. This suggests that at least part of the burden of breast cancer that we report is due to a true difference in rates, rather than solely due to the young ages of the Pacific population in New Zealand. It may be explained by cohort effects and patterns of migration, with the older Pacific women in New Zealand experiencing similar rates to the historically low rate of breast cancer in some Pacific Islands [9], although reasons for the higher rates in younger Pacific women remains unexplained.

Breast cancer has repeatedly been demonstrated to be a disease more common in affluent people [21–23]. Although in our main results, we found a higher risk in more deprived women, this was reversed in non-Māori/non-Pacific and Māori women in the weighted analysis. This latter analysis is concordant with the high rate of breast cancer reported in women in New Zealand in the highest income tertile [7]. Data from UK have found that the association between deprivation and breast cancer differs by age [24], with only small differences across deprivation groups in young women, and the largest differences in women in the screening age group. A possible explanation is the higher proportion of familial cancers in younger women [24]. Furthermore, studies suggest that deprivation may be associated with a higher risk of some breast cancer sub-types, notably ones of poorer prognosis [23]. In addition, differential associations between deprivation and breast cancer between younger and older women may be partially due to differences in detection due to differences in screening attendance, which is lower in Māori and Pacific women [25]. Of interest in our weighted analysis is the discordance in effect that we report between two measures of socio-economic position (area-level deprivation and education). This suggests that the observed results could be due to reverse causality, caused by sick people moving to poorer areas, rather than being attributable to a lifetime of exposure to adverse risks.

The very strong association that we demonstrated between number of children and breast cancer risk in Pacific women is of interest. It is possible that this is a true effect, detectable more readily in Pacific women because of the higher number of children borne by women in the most extreme category (four or more children). The mean number of children in this highest group was 5.1 in Pacific women, 4.9 in Māori, and 4.5 in non-Māori/non-Pacific women. It is also possible that this effect is stronger in Pacific women than in other women, because of differences in confounding structures between ethnic groups. For example, there was an apparent inverse association between number of children and BMI in Pacific women, as opposed to a strong positive relationship between number of children and BMI in Māori, which was weaker but still evident in non-Māori/non-Pacific women.

A recent analysis of the Women’s Health Initiative observational study found a positive association between smoking and postmenopausal breast cancer risk [26]. Among Māori women in this study, we have identified a substantial risk associated with ever having smoked, which translated to a population attributable fraction of 31 %. This is likely to be an overestimate due to residual confounding. Nevertheless, it may in part be real and may be more evident among women who smoke heavily for many years. It is also important to consider the possibility of there being a critical or sensitive period, such as smoking prior to first pregnancy [2, 27] or during pregnancy [28]. Māori have the highest rates of smoking among women worldwide, approaching 50 % [29], and start smoking heavily at a younger age than other women. A stronger effect of smoking in Māori women compared to other ethnic groups, coupled with high rates of obesity in Māori, is not consistent with a recently reported interaction between smoking and obesity and breast cancer [30], with higher risks in smokers only evident in non-obese women. Careful further analysis of this is warranted, to disentangle possible confounding and effect modification and to identify the duration and level of smoking that is driving the high risk in Māori women.

We did not find an association between height and breast cancer, despite clear evidence for an association from systematic reviews [31]. It is implausible that international evidence does not apply to the total New Zealand population. In our systematic review [31], it was clear that the association between height and breast cancer is less robust in case–control studies than in cohort studies. This suggests that if height is measured relatively late in life, as in this study, when women have already begun to experience age-related height loss, this is not related to breast cancer as strongly as is maximally attained height. This would suggest that height loss may be an indicator of breast cancer risk, for example if height loss is a marker of estrogen levels which affect bone loss.

The suggestion of differential effects of BMI across ethnic groups could be due to chance, as we have performed multiple significance tests in this initial report, although we have restricted these analyses to “established” risk factors. In non-Māori/non-Pacific women, the detrimental effect is clear in overweight women, but is not stronger in obese women. In Māori women, on the other hand, the excess risk appears to be restricted to obese women. Small numbers hamper the ability to look at this clearly in Pacific women. The stronger effect of WHtR than BMI is similar to that detected in cardiovascular disease [32], but this is one of the first explorations of this measure in relation to breast cancer. We recommend that future studies investigate the use of this measure; since BMI does not capture the percentage of body fat equally between ethnic groups [33], an easily interpreted measure of central obesity is useful and less prone to measurement error than WHR.

The expected association between alcohol and breast cancer risk was not seen in this study; further investigation of this is ongoing. We found some evidence of a detrimental effect of binge drinking, particularly in Māori women. This is consistent with recently reported data from the Nurses’ Health Study [3]. The rate of binge drinking in New Zealand is high and is disproportionate between ethnic groups; the age-adjusted prevalence is reported as 39 % in Māori; 31 % in Pacific, and 22 % in European women [34]. Given the excess burden of the alcohol-related harm borne by Māori, this is an important area for future research.

The lack of an association between HRT and breast cancer is contrary to that reported in prospective studies and clinical trials. A likely explanation is the crude measure of “ever use of HRT” that was used in this study; the risk is higher for combined estrogen/progesterone HRT compared to estrogen-only HRT [35] and also differs by cancer subtype [36]. The protective effect of OC use in Māori and non-Māori/non-Pacific women differs from that previously reported and did not appear to be explained by any of the variables available to us for which we adjusted. The observation that the effect was attenuated following adjustment for differential non-response indicates that the results may well be affected by residual confounding. New Zealand women have a very high use of Depo-Provera, and its use is more common among Māori than women of other ethnic groups [37]. One possibility is that the protective effect of OC use which we found could reflect a harmful effect of other hormonal contraceptive methods. This, plus the lack of an effect of OC use in Pacific women is further evidence that the effect in Māori and non-Māori/non-Pacific women is unlikely to be causal.

In summary, we have documented some expected, and other unexpected, associations between environmental factors and breast cancer risk in New Zealand; we have also found ethnic differences in exposure to risk factors and the associations of these factors with breast cancer. Despite methodological and statistical attempts to reduce the impact of selection bias on the results, we urge that these are interpreted with caution. This is the first such nationwide study to be conducted in New Zealand, with adequate numbers of women in different ethnic groups. Future studies need to ensure the use of appropriate methodologies to allow recruitment of participants from all ethnic groups. Most promising avenues for future research are likely to be investigations into the detrimental effects of smoking and binge drinking in relation to breast cancer risk. Reducing the burden of breast cancer in New Zealand is likely to require different approaches for different ethnic groups.
